# Risk Factors Associated With Steroid-Induced Ocular Hypertension and Glaucoma in Patients With Vernal Keratoconjunctivitis in a North Indian Population: A Multicenter Study

**DOI:** 10.7759/cureus.89972

**Published:** 2025-08-13

**Authors:** Suneeta Dubey, Rakesh Shakya, Julie Pegu, Navjot Ahluwalia, Tanima Bansal, Madhu Bhadauria, Lokesh Chauhan, Ken Nischal

**Affiliations:** 1 Glaucoma, Dr. Shroff's Charity Eye Hospital, New Delhi, IND; 2 Ophthalmology, Sadguru Netra Chikitsalaya, Shri Sadguru Seva Sangh Trust, Chitrakoot, IND; 3 Glaucoma, ICARE Eye Hospital, Noida, IND; 4 Ophthalmology, Regional Institute of Ophthalmology, Sitapur Eye Hospital, Sitapur, IND; 5 Ophthalmology, University of Pittsburgh Medical Center (UPMC) Children's Hospital of Pittsburgh, Pittsburgh, USA

**Keywords:** blindness, public health, steroid-induced glaucoma, steroid-induced ocular hypertension, vernal keratoconjunctivitis (vkc)

## Abstract

Introduction

Vernal keratoconjunctivitis (VKC) is a chronic allergic condition of the eye that often requires long-term steroid use for symptom control. However, prolonged or unsupervised steroid use can lead to serious complications like steroid-induced ocular hypertension (SIOHT) and steroid-induced glaucoma (SIG). Identifying patients at risk for these complications is crucial for timely intervention and preserving vision.

Objectives

This study aims to (1) identify and evaluate both steroid-related and non-steroid-related risk factors associated with SIOHT and SIG in patients with VKC and (2) compare clinical characteristics, including the type of steroid used, duration of use, and visual outcomes such as need for surgery and vision loss. Additionally, the study explores differences between urban and rural populations in terms of disease presentation, management, and outcomes.

Methods

A multicentric retrospective proforma-driven chart review conducted in four centers from April 2019 to March 2020 analyzed patient records for steroid use, urban/rural settings, family history of glaucoma, allergies, clinical features, and treatments.

Results

In a study of 2,360 VKC patients, 4.7% exhibited SIOHT and/or SIG. SIG and SIOHT prevalence was 53.5% and 46.5%, with mean presentation ages of 17.1 ± 5.9 and 16.4 ± 6.0 years, respectively. Topical dexamethasone (50%) was the most used steroid. Urban-rural disparities in age and steroid usage were significant. Successful management involved steroid withdrawal (10.3% eyes), anti-glaucoma medications (62.9% eyes), and surgery (24.9% eyes). Urban areas showed higher limbal VKC rates (23%) than rural areas (7.9%) (p = 0.01). Myopia was observed in 26.5% of SIG/SIOHT eyes, while hyperopia was observed in 2.8% of cases. Additionally, 29 rural and 11 urban patients had a history of allergy (p = 0.1). The findings highlight demographic variations, steroid implications, and management strategies in VKC patients with SIG and SIOHT.

Conclusion

The study highlights the significant impact of glaucoma in VKC patients, revealing that 25% require surgery. Urban areas show higher limbal VKC rates, possibly linked to air pollution. Surprisingly, even milder steroids may induce SIG and SIOHT. The results have broad relevance, as they stem from the genetic and anthropological traits of North Indian populations.

## Introduction

Vernal keratoconjunctivitis (VKC) is a chronic, bilateral allergic external eye disease mostly affecting children and young adults. Due to the chronic nature of the disease, 85% of patients with VKC use corticosteroids during the course of treatment [1]. The unmonitored and prolonged use of corticosteroids predisposes the eye to visually debilitating sequelae such as cataract and steroid-induced glaucoma (SIG) [2].

The reported prevalence of SIG in VKC patients is between 2% and 8.89% [3-7]. Sen et al. found that 19% of the patients in rural areas of India who had used steroids without supervision developed SIG [4]. SIG was found to be the most common complication in 8.89% of the patients, necessitating regular monitoring of intraocular pressure (IOP) and a change in the treatment regimen [7]. SIG is now increasingly recognized as a significant cause of visual disability in patients with VKC, with multiple studies highlighting the critical role of both the type and duration of steroid use in its development [4,5,8]. Ang et al. found clinical factors like mixed form of VKC, higher recurrences per year, and limbal vascularization with corneal involvement to be associated with greater ocular hypertensive response to steroids [9]. Gupta et al. [8] suggested that a possible reason for the increased incidence of SIG could be due to the increased incidence of VKC because of environmental factors, including an increase in air pollution [10].

Studies of SIG in VKC in the Indian population have focused on the types of steroids, potency, and duration of steroid use [1,4,5,8]. However, none of these studies assessed the association of non-steroid-related risk factors like myopia, systemic allergic association (rhinitis, atopic dermatitis, and asthma), family history of glaucoma, and environment with VKC. Our study aims to analyze the extent of associated morbidity in VKC patients; identification of risk factors for developing steroid-induced ocular hypertension (SIOHT) and SIG, such as family history, refractive status, and rural/urban background of the subject; and the final clinical outcome of the disease.

This is the first multicentric study, to the best of our knowledge, that aims to assess both steroidal and non-steroidal risk factors of SIG in VKC. Our study also aims to evaluate the differences in clinical features between urban and rural patients with SIG/SIOHT and VKC, given that patients in rural areas have limited access to eye care and are more likely to seek care from pharmacies and homeopathic and general practitioners [11] and corticosteroids remain the mainstay of their treatment because of the prompt symptomatic relief provided [12-14].

Recent advances in anthropology and genomics reveal that most Indian groups trace their ancestry to a blend of two genetically distinct populations: Ancestral North Indians (ANI) linked to Central Asians, Middle Easterners, Caucasians, and Europeans [15] and Ancestral South Indians (ASI) unique to the Indian subcontinent. Given the genetic heterogeneity among Indian populations, differences in steroid responsiveness and immune-mediated ocular diseases may be partially influenced by ancestry. This heterogeneity suggests that studies on North Indian populations may have broader global health implications, as they are more generalizable to populations beyond the Indian subcontinent, including Euro-Americans.

## Materials and methods

Patient and public involvement

Recruitment of participants and baseline assessment were conducted through a retrospective chart review. Patients or members of the public were not involved in the design, conduct, reporting, or dissemination of this research. The study had ethical approval from the Institutional Ethics Committee (letter no. IRB/2020/Oct/61), aligning with the Declaration of Helsinki guidelines.

Participants in the study were selected based on IOP measurements, either showing IOP greater than 21 mmHg on two or more occasions or IOP below 21 mmHg while on anti-glaucoma medications (AGMs). The subjects were classified into two groups: SIOHT and SIG. Individuals in the SIOHT group had IOP above 21 mmHg measured by Goldmann applanation tonometry (GAT), but without any signs of glaucomatous optic disc damage. They also had at least two reliable and normal visual field tests, defined by a pattern standard deviation (PSD) within the 95% confidence interval and a glaucoma hemifield test (GHT) result within normal limits [16]. On the other hand, the SIG group consisted of patients who had glaucomatous changes in the optic nerve head-such as a vertical cup-to-disc (CD) ratio exceeding 0.7:1, asymmetry between eyes greater than 0.2, or signs like localized notching or neuroretinal rim thinning-assessed stereoscopically using a Volk 90D lens (Volk Optical, Inc., Mentor, OH, US) by an experienced examiner.

Additionally, patients with SIG had visual field defects consistent with glaucoma (Hodapp-Parrish-Anderson criteria) and IOP above 21 mmHg at diagnosis [16]. Those with congenital, developmental, or secondary glaucomas or a history of eye surgery were excluded. We recorded demographic details (age, gender, and urban/rural residence), systemic allergies, family history of glaucoma, and details of steroid and AGM use (type, duration, and source). Eye evaluations included refraction, visual acuity, IOP, gonioscopy, lens and optic nerve status, and visual fields. Treatment course and outcomes-both medical and surgical-were documented for all patients.

Steroids were categorized based on their potency: high potency (e.g., dexamethasone 0.5% and betamethasone 0.2%), moderate potency (e.g., prednisolone acetate 1%), low potency (e.g., loteprednol and fluorometholone 0.1%), and “others” when the exact type was unknown. Additionally, VKC was classified into palpebral (characterized by papillae on the upper conjunctiva), limbal (where gelatinous deposits or papillae were mainly at the limbus), or mixed forms when features of both were observed. Visual impairment was defined as blindness when the best corrected visual acuity in the better-seeing eye was worse than 3/60 or the visual field was constricted to less than 10 degrees.

Statistical analysis was conducted using IBM SPSS version 23 (IBM Corp., Armonk, NY, US). Continuous variables were presented as the mean ± standard deviation, and categorical variables as percentages. Non-parametric comparisons were made using the Kruskal-Wallis and Mann-Whitney U tests, while the Chi-squared test was used for categorical variables. A p-value ≤ 0.05 was considered statistically significant. Missing data were handled using listwise deletion for analyses involving variables with incomplete records. The proportion of missing data has been indicated for transparency in the relevant tables.

## Results

A total of 2,360 patients with VKC were analyzed during the study period: 112 patients (4.7% of total VKC patients) had either SIG or SIOHT in at least one eye, with 213 eyes affected and included in the study. A total of 114 of these 213 eyes (53.5%) had SIG, and 99 (46.5%) eyes had SIOHT (Table 1).

**Table 1:** Demographic Profile, Steroid Usage Patterns, and VKC Classification at Presentation Statistical comparisons not applicable. “Not available” indicates missing data due to incomplete documentation in the patient record. Percentages and total numbers reflect available data for each variable *Variables that were further analyzed for statistical association with the development of SIG and SIOHT in the study cohort SIG: steroid-induced glaucoma; SIOHT: steroid-induced ocular hypertension

Total patients		213 eyes of 112 patients	
Age at presentation		16.9 ± 5.9 years	
Age of onset of vernal keratoconjunctivitis (VKC)		12.1 ± 4.7 years	
Variable	Category	Number	Percentage
Gender	Male	84	75
	Female	28	25
Location	Rural	90	80.4
	Urban	22	19.6
History of allergy	Asthma	2	1.8
	Rhinitis	9	8
	Skin allergy	11	9.8
	Nil	90	80.4
Family history of allergy	Asthma	2	1.8
	Glaucoma	7	6.3
	Rhinitis	5	4.5
	Skin allergy	3	2.7
	VKC	1	0.9
	Nil	94	83.0
Steroid used*	Dexamethasone	104	49.3
	Betamethasone	27	12.8
	Prednisolone	40	19
	Loteprednol	15	7.1
	Fluorometholone	13	6.2
	Others	12	5.7
Steroid prescribed by	Pharmacist	18	16.1
	Ophthalmologist	37	33
	Self/over-the-counter/non-medical personnel	53	47.3
	Not known	4	3.6
Duration of steroid use	≤1 month	10	8.9
	>1-6 months	46	41.1
	>6 months	54	48.2
	Not available	2	1.8
Eye*	OD	105	49.8
	OS	106	50.2
Anti-glaucoma medications (AGMs) at presentation*	Yes	83	36.7
	No	143	63.3
	Not available	7	3.3
If yes, number of AGMs*	One	23	10.9
	Two	12	5.7
	Three	20	9.5
	Four	9	4.3
	Five	3	1.4
Type*	Steroid-induced glaucoma	114	54.0
	Steroid-induced ocular hypertension	96	45.5
Management*	Withdraw steroid	20	9.5
	AGMs	134	63.5
	Surgical	53	25.1
	Lost to follow-up	4	1.9
Type of VKC*	Bulbar/limbal	23	10.9
	Conjunctival	59	28
	Mixed	123	58.3
	Not available	6	2.8

The mean age of presentation of patients with SIG and SIOHT was 17.1 ± 5.9 years (5-29 years) and 16.4 ± 6.0 years (5-28 years), respectively (p = 0.38). The average age of presentation of the study population of rural background was 17.4 ± 5.8 years, and that of urban background was 14.1 ± 5.9 years (p = <0.001).

Mean IOP at presentation in patients with SIG was 35.1 ± 13.2 mmHg, and that of SIOHT was 28.0 ± 7.1 mmHg (p = <0.001). We did not find significant differences in presenting IOP between rural (32.2 ± 11.7 mmHg) and urban (29.6 ± 10.0 mmHg) populations (p = 0.17). The most common form of VKC in the study population was the mixed type (n = 123 eyes; 57.7%), followed by tarsal (n = 61; 28.6%) and limbal (n = 23; 10.8%) (Table 2).

**Table 2:** Comparison Between Rural and Urban VKC Patients With Respect to Demographics, Clinical Profile, and Steroid Use Statistical tests used: Chi-squared test for categorical variables; Mann–Whitney U test for continuous variables. Significance threshold: p < 0.05. “Not available” indicates missing data due to incomplete documentation in the patient record. Percentages and total numbers reflect available data for each variable CD: cup-to-disc

Rural versus urban	Rural	Urban	p-value
Age of onset of vernal keratoconjunctivitis (VKC)	12.5 ± 4.6 Y (range: 3-23 Y)	10.5 ± 4.1 Y (range: 4-20 Y)	0.002
Age at presentation	17.5 ± 5.8 Y (range: 5-29 Y)	14.1 ± 5.9 Y (range: 6-25 Y)	0.001
Intraocular pressure (IOP) at presentation	32.2 ± 11.7 mmHg	29.5 ± 9.9 mmHg	0.17
History of allergy			
Yes	16 (17.7%)	06 (27.2%)	0.37
No	74 (82.3%)	16 (72.7%)	
Type of steroid			
Dexamethasone	98 (53.6%)	15 (34.9%)	<0.01
Betamethasone	26 (14.2%)	2 (4.7%)	
Prednisolone	37 (20.2%)	6 (14%)	
Loteprednol	13 (7.1%)	4 (9.3%)	
Fluorometholone	7 (3.8%)	6 (14%)	
Others	2 (1.1%)	10 (23.3%)	
Duration of steroid use			
≤1 month	40 (21.9%)	10 (23.3%)	<0.01
>1 month to 6 months	110 (60.1%)	11 (25.6%)	
>6 months	33 (18%)	22 (51.2%)	
Steroid prescribed by			
Pharmacist	21 (14.1%)	4 (9.8%)	0.02
General ophthalmologist	75 (50.3%)	13 (31.7%)	
Self/non-medical person	53 (35.6%)	24 (58.5%)	
Vision at presentation			
6/6-6/12	130 (71.4%)	27 (62.8%)	0.53
6/18-6/60	22 (12.1%)	7 (16.3%)	
<6/60	30 (16.5%)	9 (20.9%)	
History of anti-glaucoma medications (AGMs) at presentation			
Yes	70 (39.5%)	13 (31%)	0.37
No	107 (60.5%)	29 (69%)	
If yes, number of AGMs	2.3 ± 1.2	2.6 ± 0.9	0.32
Type of VKC			
Conjunctival	51 (28.8%)	14 (32.6%)	0.005
Bulbar/limbal	13 (7.3%)	10 (23.3%)	
Mixed	113 (63.8%)	19 (44.2%)	
CD ratio at presentation			
0.3-0.5	82 (44.8%)	17 (41.5%)	0.64
0.6-0.8	43 (23.5%)	8 (19.5%)	
>0.8	58 (31.7%)	16 (39%)	
Classified as			
Steroid-induced ocular hypertension	86 (47%)	19 (44.2%)	0.86
Steroid-induced glaucoma	97 (53%)	24 (55.8%)	

In the study, 19.6% had allergies (9.8% skin, 8% rhinitis, and 1.8% asthma). A glaucoma family history was noted in 6.3%. Allergy history in rural (17.1%) and urban (25.6%) areas showed a non-significant difference (p = 0.20).

The median duration of steroid use in the SIG population was six months (range: 2-84), and that of SIOHT was 12 months (range: 1-84), with a statistically significant (p = <0.001) difference. In the study population, 50% of the eyes had used dexamethasone, 18.8% prednisolone, 12.5% betamethasone, 7.1% loteprednol, and 6.3% fluorometholone. In the rural population, almost half the eyes were on dexamethasone (52.4%), followed by prednisolone (20%), betamethasone (14.7%), loteprednol (7.6%), and fluorometholone (4.1%). In the urban cohort, 34.9% used dexamethasone, 14% each used prednisolone and fluorometholone, 9.3% used loteprednol, and 4.7% used betamethasone, while other drops were used by 23.3% of the patients.

In our study, 47.3% of patients used steroids either self-prescribed or over-the-counter purchased. Almost a third of the patients, 30.4 %, were prescribed drops by an ophthalmologist and 16.1% by the pharmacist, while 2.7% were prescribed by non-medical personnel. In rural areas, 54% used the drops by over-the-counter purchases, 31.7% were prescribed drops by ophthalmologists, and 14.1% were prescribed by pharmacists. In urban areas, 37.1% used the drops by over-the-counter use, 51.4% were prescribed drops by ophthalmologists, and 11.4% were prescribed by pharmacists.

The average number of patients on AGM at presentation in the SIG population was 52.6%, and the SIOHT population was 16.3%. The average number of AGMs at presentation in the SIG group was 2.8 ± 1.2 and 1.3 ± 0.9 in the SIOHT group, respectively. Overall, 22 (10.3%) eyes were managed by withdrawing steroids, 134 (62.9%) eyes by AGM, and 53 (24.9%) by performing surgeries. All SIOHT patients were managed either by withdrawal of steroids or by AGM. The mean IOP at the last follow-up visit was 15.4 ± 7 mmHg. The presenting and final visual acuities in SIG and SIOHT are given in Table 3. The average CD ratio in the study population in SIG and SIOHT at presentation and final follow-up is also shown in Table 3.

**Table 3:** Demographic and Clinical Outcome Comparison Between Steroid-Induced Glaucoma (SIG) and Steroid-Induced Ocular Hypertension (SIOHT) Groups Statistical tests used: Chi-squared test for categorical variables; Mann–Whitney U test for continuous variables. Significance threshold: p < 0.05 CD: cup-to-disc

	SIG	SIOHT	p-value
Age of onset of vernal keratoconjunctivitis (VKC)	11.9 ± 4.4 Y (range: 3-23 Y)	12.3 ± 5 Y (range: 3-21 Y)	0.69
Age at presentation	17.4 ± 5.9 Y (range: 5-29 Y)	16.4 ± 5.8 (range: 5-26 Y)	0.38
Intraocular pressure (IOP) at presentation	35.1 ± 13.1 mmHg	28.1 ± 7.1 mmHg	<0.01
History of allergy			
Yes	13 (22.4%)	9 (17%)	0.63
No	45 (77.6%)	44 (83%)	
Type of steroid			
Dexamethasone	71 (62.3%)	33 (34.4%)	<0.01
Betamethasone	8 (7%)	18 (18.8%)	
Prednisolone	20 (17.5%)	20 (20.8%)	
Loteprednol	2 (1.8%)	13 (13.5%)	
Fluorometholone	2 (1.8%)	11 (11.5%)	
Others	11 (9.6%)	1 (1%)	
Duration of steroid use			
≤1 month	0	10 (18.9%)	0.001
>1 month to 6 months	23 (58.9%)	22 (41.5%)	
>6 months	33 (41.1%)	21 (39.6%)	
Steroid prescribed by			
Pharmacist	16 (28.1%)	20 (40%)	0.32
General ophthalmologist	9 (15.8%)	9 (18%)	
Self/non-medical person	32 (56.1%)	21 (42%)	
Vision at presentation			
6/6-6/12	57 (50.4%)	90 (93.8%)	<0.01
6/18-6/60	23 (20.4%)	5 (5.2%)	
<6/60	33 (29.2%)	1 (1%)	
History of anti-glaucoma medications (AGMs) at presentation			
Yes	60 (53.6%)	16 (17.4%)	<0.01
No	52 (46.4%)	76 (82.6%)	
If yes, number of AGMs	2.5 ± 1.2	1.6 ± 0.8	0.9
Type of VKC			
Conjunctival	29 (26.4%)	30 (31.9%)	0.15
Bulbar/limbal	9 (8.2%)	14 (14.9%)	
Mixed	72 (65.5%)	50 (53.2%)	
CD ratio at presentation			
0.3-0.5	7 (6.2%)	85 (88.5%)	<0.01
0.6-0.8	38 (33.9%)	11 (11.5%)	
>0.8	67 (59.8%)	0	

Sixty-six eyes (30.9%) of SIG and 38 eyes (17.8%) of SIOHT were diagnosed with refractive error (p = 0.003). Among eyes with refractive error, the proportion of myopic eyes was distributed evenly among SIG (n = 35, 53%) and SIOHT (n = 21, 55.2%) patients (p = 0.84). A total of 56 (26.5%) eyes were diagnosed with myopia, six (2.8%) eyes with hyperopia, and 42 (19.9%) eyes with astigmatism. There was a higher proportion of myopic eyes in the urban population compared to the rural population (n = 20, 46.5% versus n = 38, 22.6%) (p = 0.005).

To identify independent risk factors for the development of SIG as opposed to SIOHT, we performed binary logistic regression analysis. Variables included were age at presentation, duration of steroid use, type of steroid used (high/moderate vs. low potency), refractive error (presence of myopia), urban vs. rural residence, and presence of systemic allergy.

Table 4 shows the results of the multivariate logistic regression model. Longer duration of steroid use (>6 months), use of high-potency steroids (dexamethasone or betamethasone), and urban residence were found to be significantly associated with higher odds of developing SIG.

**Table 4:** Multivariate Logistic Regression Analysis of Risk Factors for Steroid-Induced Glaucoma Logistic regression model used. Statistical significance defined as p < 0.05

Variable	Odds ratio (OR)	95% confidence interval	p-value
Duration of steroid use >6 mo	3.21	1.76–5.85	<0.001
High-potency steroid use	2.89	1.55–5.39	0.001
Urban residence	2.01	1.01–3.99	0.045
Myopia	1.34	0.74–2.45	0.33
Age at presentation (>15 yrs)	1.18	0.65–2.15	0.59
Systemic allergy present	0.96	0.44–2.08	0.91

## Discussion

SIG is an important cause of avoidable blindness in patients with VKC. The indiscriminate and often unmonitored use of topical steroids is fast becoming an important public health concern. Affecting the young can lead to many blind years, with a significant effect on the individual’s quality of life, as well as the economics of healthcare. VKC poses a significant burden on the lives of patients and their families [17,18]. Greater awareness among clinicians is essential for early diagnosis and treatment and to prevent potential sight-threatening complications [17]. Wadhwani et al. reported that 69.2% of caregivers were not aware of the symptoms of VKC, and 83% of caregivers were unaware of the side effects of eye drops used [18].

To address the primary objective of identifying risk factors for SIG in VKC patients, we performed multivariate analysis. The findings confirm that chronic steroid use, especially high-potency preparations like dexamethasone and betamethasone, is a significant independent risk factor for SIG. Furthermore, urban residency was associated with greater odds, possibly due to a higher prevalence of limbal VKC or increased unsupervised access to potent steroids.

Interestingly, although myopia has been hypothesized as a risk factor for steroid responsiveness, it was not found to be statistically significant in our multivariate analysis. These results strengthen the need for early screening and controlled steroid prescription protocols in urban clinical settings.

VKC is known to primarily affect pre-teen boys, resolving spontaneously with or after puberty [17], but may persist in some beyond 20 years of age [6]. The mean age of presentation of SIG in our study population was 17.1 ± 5.9 years (5-29 years), and that of SIOHT was 16.4 ± 6.0 years (5-28 years). Various authors have variably reported the mean age of 14.1 ± 3.8 years [4], 12 years [5], and 12 ± 4.8 years [8]. We report a relatively higher age of presentation of SIG and SIOHT compared to the above studies, likely because we included all patients diagnosed with VKC regardless of age, unlike other previous reports [4-8], which set an age limit. Our study highlights that SIG in VKC can present late, up to 29 years of age, confirming previous reported findings [5] and emphasizing the chronic nature of the disease.

The rural population in our study presented at a significantly older age than the urban group (p = 0.001), likely reflecting delayed access to healthcare. The overall male-to-female ratio was 3:1, consistent with previous studies [4,7]. While VKC is more common in boys, this disparity may also reflect underlying gender bias in healthcare access, where girls’ symptoms are more often overlooked in some regions of India [19].

The mean age of presentation with VKC in our study population was 12.0 ± 4.7 years (range 3-23 years), with significantly earlier onset in the urban group (10.0 ± 4.1 years) compared to the rural group (12.5 ± 4.8years) (p < 0.001), most likely due to easier access to healthcare although exposure to air pollution in the urban setting cannot be entirely ruled out.

The study found significantly longer steroid use in the SIG (20.9 ± 20.8 months) than SIOHT (10.2 ± 14.8 months) groups (p < 0.001), indicating potential progression from SIOHT to SIG due to chronic steroid use. Rural (15.2 ± 18.7 months) and urban (19.2 ± 19.7 months) populations showed non-significant differences in duration (p = 0.23).

The most common VKC type in our cohort was the mixed form (57.7%), followed by palpebral (28.6%) and limbal (10.8%), consistent with previous studies [4,5,7]. We observed a statistically significant difference in VKC type distribution between rural and urban populations (p = 0.01), with limbal VKC more frequently seen in urban patients (23.3% vs. 7.9%). This may reflect environmental factors such as greater exposure to air pollution. Notably, no significant difference in VKC subtype distribution was found between the SIG and SIOHT groups (p = 0.12).

On analyzing the types of steroids for the study population, it was found that 50% of the eyes used dexamethasone, 18.8% prednisolone, 14% betamethasone, 7.1% loteprednol, and 6.3% fluorometholone. Dexamethasone was the most common steroid used in both groups, but its use was significantly higher in the rural population (52.4%) compared to the urban population (34.9%) (p = 0.001). A higher percentage of fluorometholone use was seen in the urban population (14%) compared to the rural population (4.1%). This confirms previous reports [5,7], especially for dexamethasone, which is cheap and freely available over the counter in India [20]. Our data confirm that low-potency topical steroids can still cause a significant rise in IOP and SIG.

In our study, 10.3% of eyes had steroids withdrawn, 62.9% required AGMs, and 24.9% needed surgery. All SIOHT cases were managed by steroid withdrawal or AGMs due to varying topical steroid use duration. Prolonged use may contribute to irreversible trabecular meshwork damage, resulting in persistent IOP elevation requiring medication or surgery. This underscores the importance of timely intervention and careful monitoring in cases of SIOHT [21-25].

Discontinuing steroids can reverse the IOP elevation in subjects who have used topical steroids for less than eight weeks [9]. Senthil et al., on the contrary, reported that discontinuation of steroids helped to control IOP in only 2% patients, and the overwhelming majority required either AGM (66%) or surgery (34%) for IOP control [5].

Various authors have reported that 16% to 64% of eyes require surgery for IOP control [7,22,25], while medical treatment has been reported to be more effective in patients on systemic steroids as compared to topical steroids [7,21,22]. Other associations for the need for surgery include longer duration of steroid use, higher peak IOP and greater increase in IOP from baseline [24], higher baseline IOP (IOP > 45 mmHg), age less than 20 years (representing eyes with VKC and SIG), and eyes with greater glaucomatous optic neuropathy (CD ratio: 0.87 compared with 0.71) [22]. In our study, almost 60% of patients presented with a CD ratio of 0.8 or more in the SIG group, suggestive of advanced glaucoma, and 34% of the patients had a CD ratio between 0.6 and 0.8.

In our study, 26.5% eyes were diagnosed with myopia and only 2.8% eyes with hyperopia. However, the proportion of myopic eyes was distributed evenly among SIG (n = 35, 53%) and SIOHT (n = 21, 55.2%) patients. It has been reported that there is a greater risk of corticosteroid response in patients with high myopia. Also, in our study, the prevalence of myopia (26.5%) is higher than previously reported studies among school-going children from India.

As many as 23.6% of the eyes in the SIG group had vision less than 6/60 at the last follow-up in our study. There were no eyes in the SIOHT group with vision less than 6/60. Previously, authors have variably reported the incidence of blindness ranging from 27% to 43.4% [5,7]. Senthil et al. reported that 43.4% of eyes in their case series were blind at presentation, with three-fourths bilaterally blind and one-fourth blind in one eye [5]. These variations may be attributed to the variable severity of glaucoma and the age of presentation in the various studies. Also, given that Senthil et al. reported the incidence of blindness from a tertiary care center, their data may be subject to referral bias, with more serious patients being referred to the center. Our data set may be a more accurate real-world picture, since the multiple centers not only cater to referred patients but also see patients de novo. That said, it stands to reason to state that the patients with a higher CD ratio at presentation and a longer duration of steroid use have a higher risk of blindness.

This study has a few limitations because of its retrospective design. Most of the information on steroid use-like the type, duration, and where it was obtained-came from what patients shared during their clinic visits. While these details were recorded in patient charts, they may not always be accurate, especially when prescriptions or packaging were not available. Some data were also missing due to incomplete documentation, which we have now reported and accounted for in our analysis. The assessment of glaucoma severity was done by experienced doctors at different centers, but since evaluations were not done by a single person or blinded manner, there may be some differences in how optic nerve changes were judged. We used logistic regression to study risk factors, but not all statistical checks were included-such as interactions between variables or testing for overlapping effects (collinearity). We have now added measures of model fit, but results like the association with urban residence should still be interpreted carefully. Lastly, our findings are most applicable to the North Indian population studied and may not directly apply to other regions. We have also adjusted our interpretation of myopia since it was not a statistically significant risk factor in our analysis.

The use of steroid medications, whether judicious or indiscriminate, carries a serious threat to vision. SIG and SIOHT are both sight-threatening complications of the use of steroids for VKC. General physicians and ophthalmologists must be aware of these sight-threatening side effects, emphasizing the need for regular eye pressure monitoring. Training in the use of steroid-sparing anti-allergic medications and implementing an escalation matrix for VKC treatment are vital. Public awareness, particularly among parents of young children, about the dangers of self-medication and indiscriminate steroid use is mandatory. Strict adherence to government regulations, including the prohibition of over-the-counter sales of steroid medications, is essential. These measures are pivotal in averting avoidable blindness caused by SIG in VKC-afflicted children. Notably, this study's findings apply to diverse populations, encompassing Middle Easterners, Central Asians, and Europeans, underlining the global relevance of these crucial precautions.

## Conclusions

The study highlights the significant impact of glaucoma in VKC patients, revealing that 25% require surgery. Urban areas show higher limbal VKC rates, possibly linked to air pollution. Surprisingly, even milder steroids may induce SIG and SIOHT. The results have broad relevance, as they stem from the genetic and anthropological traits of North Indian populations.
